# Preterm birth and future risk of maternal cardiovascular disease – is the association independent of smoking during pregnancy?

**DOI:** 10.1186/s12884-015-0571-7

**Published:** 2015-07-04

**Authors:** Anh D. Ngo, Jian Sheng Chen, Gemma Figtree, Jonathan M. Morris, Christine L. Roberts

**Affiliations:** Clinical and Population Perinatal Health Research, Kolling Institute, University of Sydney at Royal North Shore Hospital, St Leonards, New South Wales NSW 2065 Australia; Department of Cardiology, Royal North Shore Hospital, St Leonards, New South Wales NSW 2065 Australia

**Keywords:** Cardiovascular disease, Preterm birth, Record linkage, Hospitalization, International classification of disease

## Abstract

**Background:**

While the association of preterm birth and the risk of maternal cardiovascular disease (CVD) has been well-documented, most studies were limited by the inability to account for smoking during pregnancy – an important risk factor for both preterm birth and CVD. This study aimed to determine whether the increased future risk of maternal cardiovascular disease (CVD) associated with preterm birth is independent of maternal smoking during pregnancy.

**Methods:**

A population-based record linkage study of 797,056 women who delivered a singleton infant between 1994 and 2011 in New South Wales, Australia was conducted. Birth records were linked to the mothers’ subsequent hospitaliation or death from CVD. Preterm births were categorised as late (35-36 weeks), moderate (33–34 weeks), or extreme (≤32 weeks); and as spontaneous or indicated. Cox proportional hazard regression was used to estimate adjusted hazard ratios (AHR) [95 % CI].

**Results:**

During the study period, 59,563 women (7.5 %) had at least one preterm birth. After adjustment for CVD risk factors other than smoking, AHR [95 % CI] of CVD among women who ever had a preterm birth was 1.78 [1.61–1.96]. Associations were greater for extreme (AHR = 1.98 [1.63–2.42]) and moderate (AHR = 2.06 [1.69–2.51]) than late preterm birth (AHR = 1.63 [1.44–1.85]), for indicated (AHR = 2.04 [1.75–2.38]) than spontaneous preterm birth (AHR = 1.65 [1.47–1.86]), and for having ≥ two (AHR = 2.29[1.75–2.99]) than having one preterm birth (AHR = 1.73[1.57–1.92]). A further adjustment for maternal smoking attenuated, but did not eliminate, the associations. Smoking during pregnancy was also independently associated with maternal CVD risks, with associations being stronger for mothers who smoked during last pregnancy (AHR = 2.07 [1.93–2.23]) than mothers who smoked during a prior pregnancy (AHR = 1.64 [1.41–1.90]).

**Conclusions:**

Associations of preterm birth and maternal CVD risk are independent of maternal smoking during pregnancy. This underscores the importance of smoking cessation in reducing CVD and suggests that a history of preterm delivery (especially if severe, indicated or recurrent) identifies women who could be targeted for CVD screening and preventative therapies.

## Background

Preterm birth is a major pregnancy complication affecting 5–18 % of deliveries worldwide [1, 2]. Growing epidemiological evidence, now synthesized in systematic reviews, has demonstrated that mothers who give birth preterm are at greater risk of developing cardiovascular disease (CVD) [3, 4]. For women with a history of giving birth preterm, the risk of CVD morbidity or death in the years following delivery is 1.2 to 4 times higher than women who had term babies. Increased risk has been reported to be greater in women with extremely preterm birth (compared to women with moderately preterm birth) [3] and in women with indicated (compared to spontaneous) preterm birth [5]. Furthermore, one study has demonstrated a linear association between the number of previous preterm births and the incidence of maternal CVD hospitalisations and morbidity in the years following pregnancy [6].

Cigarette smoking is a major modifiable risk factor for both CVD and preterm birth [1]. While the association of preterm birth and the risk of maternal CVD has been well-documented, to date only one population-based longitudinal study was able to account for the potential influences of maternal smoking [7]. However, this study was limited by the inability to control for maternal hypertensive diseases and did not differentiate the association by the onset (spontaneous or indicated) and number of preterm births. To address this knowledge gap, we conducted a population-based record linkage study of parturient women in New South Wales (NSW), Australia to examine whether the relationship of preterm birth and maternal CVD risk later in life is independent of maternal smoking during pregnancy. We hypothesized that maternal smoking during pregnancy would attenuate, but not eliminate, the association of preterm birth and maternal CVD and that the associations would persist across the preterm birth subgroups based on the severity, onset, and number of preterm births.

## Methods

### Study population and data sources

The current study is based on a cohort of women who had a singleton birth between January 1994 and December 2011 in NSW. With a population of 7 million, NSW is the most populous state in Australia with approximately one third of all Australian births. We excluded women who ever had a pregnancy complicated by chronic or pregnancy hypertension (gestational hypertension, preeclampsia, and eclampsia) during the study period, because these conditions are established risk factors for both preterm birth and later life maternal CVD [1, 8]. We also excluded women who died before the commencement of follow up, women with a CVD event prior to last birth, and women who had an event within 42 days of last birth to minimize the immediate effect of pregnancy on maternal CVD outcomes. Last birth was index birth for all women.

Four computerised datasets were linked for the present study: Perinatal Data Collection (PDC) (birth data – from 1994–2011), Admitted Patient Data Collection (APDC) (hospital data - from July 2000 to June 2012), Registrar of Births, Deaths and Marriages (RBDM) (death data - from January 1994 and December 2011), and Australian Bureau of Statistics (ABS) (cause of death data - from January 1994 and December 2007). The PDC is a statutory surveillance system of all births ≥20 weeks of gestation or ≥ 400 g birth weight in NSW, containing information on maternal characteristics, pregnancy, labour, delivery and infant outcomes that is reported by the attending clinician. APDC is a census of inpatient admissions from all public and private hospitals in NSW. It includes information on patient diagnoses and procedures documented in medical records, and coded according to the Tenth revision of the International Classification of Disease – Australian Modification (ICD10-AM). The RBDM records all deaths in NSW, while the ABS codes the cause of death according to the ICD9 (before 2000) and ICD10-AM (from 2000).

Records were linked cross-sectionally (e.g., birth to hospital records) and longitudinally to the mothers’ subsequent hospitalisation or death records to create complete obstetric and medical histories. All linkage was undertaken by the NSW Centre for Health Record Linkage [9]. Probabilistic linkage methods [10] were used to match each woman’s records based on personal information such as name, date of birth, residential address and hospital. For this study, the NSW Centre for Health Record Linkage reported high linkage quality with 3 per 1,000 false positive and <5 per 1,000 missed links [9], and the linkage proportion for maternal records is over 98 % [10]. Approval for the study was provided by NSW Population and Health Services Research Ethics Committee.

### Exposures

Gestational age (expressed as completed weeks of gestation) was based on the best clinical estimate using ultrasound examination and/or last menstruation period. Since 1990 over 94 % of pregnant women in NSW have had 2^nd^ trimester ultrasound scans [11]. Gestational age in the Perinatal Data Collection is preferentially taken from the scan reports, which were reported to have a high level of accuracy in validation studies [12–14]. Preterm births were categorised according to the severity (i.e., late (35–36 weeks of gestation), moderate (33 – 34 weeks of gestation), and extreme (20–32 weeks of gestation)), onset (spontaneous and indicated), and number (one or ≥ two). Spontaneous preterm births included those following spontaneous onset of labour or preterm premature rupture of the membranes (PPROM). Indicated preterm births included those with prelabour caesarean section or induction of labour. A woman was defined as exposed if she had at least one preterm birth. Women with more than one preterm birth were coded as having the most severe (lower gestation) preterm birth, while women with both spontaneous and indicated preterm birth were coded as having indicated preterm birth.

Women who ever smoked during pregnancy were identified from birth data (before July, 2000) or both birth and hospital data (from July, 2000) to maximize ascertainment [15]. Smoking women were classified as: those who smoked during last pregnancy and those who smoked during a prior pregnancy.

### Outcomes and follow up

The primary outcome was a first CVD event defined as hospitalisation (>42 days of last delivery) or death from any CVD: coronary heart disease (CHD) (ICD10-AM codes: I20–I25 or revascularisation procedure), the CHD subgroup - myocardial infarction (MI) (ICD10-AM codes: I21, I22, I25.2), cerebrovascular events (ICD10-AM codes: I60–I66 ; I67.0–I67.2 ; I67.4–I67.9; I68.1, I68.2, I68.8, I69, G46; G45.0-G45.2, G45.4, G45.8, G45.9), and congestive heart failure (ICD 10-AM codes: I50 ). Hospitalisations for CVD were identified, using 20 diagnosis and procedure fields in hospital records, while death from CVD as the underlying cause was ascertained from the ABS cause of death data. The secondary outcomes comprised first events for each CVD subgroup (i.e., CHD, MI, cerebrovascular events, and congestive heart failure). Validation studies show that CVD outcomes are accurately and reliably obtained from hospital data with high positive predictive values (e.g., MI: 96 %; cerebrovascular events: 93 %) [16, 17]. Follow up started at 42 days after index birth and was censored at the date of the first CVD hospitalization, date of death, or the end of the study period (30 June, 2012).

### Covariates

We used birth data (before July, 2000) or both birth and hospital data (from July, 2000) to ascertain information on other CVD risk factors and obstetric complications (dichotomised as ever versus never), including pregestational and gestational diabetes, fetal death, and small for gestational age (SGA) infant (<3^rd^ percentiles; 3- <10^th^ percentiles of infant weight by sex)[18]. Socio-demographic characteristics included maternal age at the last birth (categorised as <20; 20–35; and >35), place of birth (Australia or New Zealand, Europe or North America, Asia, and other), and socioeconomic status. Socioeconomic status was determined using the Socioeconomic Indexes for Areas (SEIFA) Relative Disadvantage developed by the Australian Bureau of Statistics and categorised into quintiles. The perinatal exposures and covariates are reliably reported with high positive predictive values (e.g., gestational diabetes: 99.7 %; pregnancy hypertension: 91.9 %) when compared with medical records [19–21].

### Statistical analysis

We first compared baseline demographic and clinical characteristics of women who ever or never had a preterm birth, using chi-square statistics. We then tested the overall association of preterm birth and maternal prenatal smoking with the first occurrence of any CVD and CVD subgroups (i.e., CHD, MI, cerebrovascular event, congestive heart failure) in three sequential Cox proportional hazard regression models. Univariate model provided crude hazard ratios (HRs). Multivariate model 1 tested included preterm birth, and all other covariates except smoking (e.g., maternal age at delivery, country of birth, socioeconomic status, number of births, pregestational and gestational diabetes, fetal death, and SGA). Multivariate model 2 included all variables in multivariate model 1 plus smoking. Interaction between maternal smoking and preterm birth was also tested by adding an interaction term in the multivariate models. Two-tailed 95 % confidence interval (CI) and *p* values were ascertained, with *p <* 0.05 regarded as significant. If HR of the interaction term was not statistically significant, the interaction term was excluded from the model. Separate sequential Cox proportional hazard regression models were then performed in the same way where preterm birth exposure was categorised according to the severity, onset and number of preterm births.

We also undertook a subgroup analysis in which we restricted the study population to index births occurring from 2001, the period when follow up was complete for all women. Furthermore, to minimize any effect of underlying cardiac diseases that may have occurred in early in life (e.g., congenital anomalies, rheumatic heart disease), we performed a subgroup analysis in which only women ≥ 35 years old at the commencement of follow up were included. The SAS 9.3 statistical package (SAS Institute Inc., Cary, NC) was used for all analyses.

## Results

**Fig. 1:**
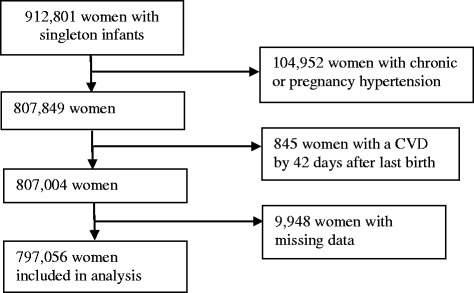
Flow chart of selection of women for analysis

Of 912,801 women who gave a singleton birth during the study period, 104,952 women (11.6 %) who ever had a pregnancy complicated by chronic hypertension (2.3 %) or pregnancy hypertension (9.3 %), and 845 women with a CVD before last birth or having a CVD event within 42 days of last birth were excluded. A further 9,948 women (1.1 %) were subsequently excluded due to missing covariate information (Fig. 1). After all exclusions, 797,056 women remained for the analysis. The median age of women at last birth was 31 years (inter-quartile range: 27–35).

The characteristics of the study population are presented in Table 1. Of 59,563 women (7.5 %) has at least one preterm birth, 35,558 (4.5 %), 10,700 (1.3 %), and 13,305 (1.7 %) ever had a late, moderate, and extreme preterm birth, respectively, while 38,435 (4.8 %) and 21,128 (2.7 %) ever had a spontaneous and indicated preterm birth, respectively, and 53,851 (6.8 %) and 5,712 (0.7 %) had one or ≥ 2 preterm births, respectively. Overall, there were 31,222 women (3.9 %) who smoked during a pregnancy before last pregnancy, and 121,467 women (15.2 %) who smoked during last pregnancy. Compared to mothers of term infants, mothers who ever had a preterm infant were more likely to be in the older age group (i.e., ≥ 35 years old), born in Australian or New Zealand, from areas of socioeconomic disadvantage, and to have had ≥ 3 births (Table 1). They were also more likely to have had a pregnancy complicated by pregestational diabetes, gestational diabetes, SGA infants, or fetal deaths, and more likely to have smoked during or before last pregnancy (Chi-square statistics: *p* < .0001 for all variables) (Table 1).

**Table 1:** Characteristics of the study participants (*n* = 797,056)

	Women with term birth (n = 737,493) n (%)	Women with preterm birth (n = 59,563) n (%)
Age group		
<20	19,146 (2.6)	1,635 (2.7)
20-34	524,443 (71.1)	40,334 (67.8)
≥35	193,904 (26.3)	17,594 (29.5)
Country of birth		
Other	56,046 (7.6)	4,348 (7.3)
Asia	110,122 (14.9)	7,526 (12.6)
Australia/NZ	515,753 (69.9)	44,122 (74.1)
Europe/North America	55,572 (7.6)	3,567 (6.0)
SEIFA index		
1^st^ quintile (most advantaged)	145,600 (19.7)	9,761 (16.4)
2^nd^ quintile	137,434 (18.6)	10,205 (17.2)
3^rd^ quintile	152,245 (20.6)	12,831 (21.5)
4^th^ quintile	147,983 (20.2)	12,475 (20.9)
5^th^ quintile (most disadvantaged)	154,231(20.9)	14,291 (24.0)
Number of births		
1	220,409 (29.9)	13,284 (22.3)
2	291,368 (39.5)	21,461 (36.0)
≥3	225,716 (30.6)	24,818 (41.7)
Smoking during pregnancy		
Never	602,684 (81.7)	41,683 (70.0)
Smoking before last pregnancy	26,914 (3.7)	4,308 (7.2)
Smoking during last pregnancy	107,895 (14.6)	13,572 (22.8)
Gestational diabetes		
Never	690,275 (93.6)	54,163 (90.9)
Ever	47,218 (6.4)	5,400 (9.1)
Diabetes		
No	734,281 (99.6)	58,768 (98.7)
Yes	3,212 (0.4)	795 (1.3)
Having a fetal death		
Never	734,399 (99.6)	53,539 (89.9)
Ever	3,094 (0.4)	6,024 (10.1)
Having an SGA infant		
Never	631,749 (85.7)	48,769 (81.9)
3- <10^th^ percentiles	75,580 (10.2)	7,131 (12.0)
<3^rd^ percentiles	30,164 (4.1)	3,663 (6.1)

The median follow-up time was 7.5 years (inter-quartile range: 3.0–13.0) encompassing 6,533,094 person-years at risk, after taking into account 3,201 deaths (0.4 %). During the follow-up period, 4,046 women developed a first CVD event comprising 4,010 hospitalisations and 36 deaths. The number of events for specific CVD subgroups included: 1,968 with CHD; 720 with MI; 1,848 cerebrovascular events; and 1,446 with congestive heart failure. The median age at the first event for CVD was 42 years old. The overall crude incidence rate for a first CVD event was 62 per 100,000 person years at risk.

### Association of preterm birth and maternal smoking with CVD

Cox proportional hazard regression analyses showed that both preterm birth and maternal smoking were independently and significantly associated with the first occurrence of CVD (*p* < 0.001) (Table 2) with no statistical evidence of interaction between these 2 conditions (*P* ≥ 0.27). Specifically, in multivariate model 1 adjusted for CVD risk factors other than smoking, compared to mothers of term infants, AHR [95 % CI] of CVD among mothers with any preterm birth was 1.78 [1.61 – 1.96] (Table 2). AHRs [95 % CI] of CVD among mothers of late, moderate, and extreme preterm infants were 1.63 [1.44–1.85], 2.06 [1.69–2.51], and 1.98 [1.63–2.42], respectively, while AHRs [95 % CI] of CVD among mothers with spontaneous and indicated preterm births were 1.65 [1.47–1.86] and 2.04 [1.75–2.38], respectively. AHRs [95 % CI] of CVD among mothers with one and ≥ 2 preterm births were 1.73 [1.57–1.92] and 2.29 [1.75–2.99], respectively. A further adjustment for maternal smoking (multivariate model 2) attenuated the strength of these associations. Similar associations were observed for the specific CVD subgroups: CHD, MI, cerebrovascular event, and congestive heart failure (Tables 3, 4, 5 and 6).

**Table 2:** Association of preterm birth and smoking with maternal cardiovascular disease (*n* =4,046)

	Univariate			Multivariate 1			Multivariate 2		
	HR	95 % CI		HR	95 % CI		HR	95 % CI	
Term birth	Ref			Ref			Ref		
Any preterm birth	2.01	1.83	2.20	1.78	1.61	1.96	1.65	1.50	1.83
Preterm birth by severity									
Late preterm birth	1.79	1.59	2.03	1.63	1.44	1.85	1.53	1.35	1.74
Moderately preterm birth	2.36	1.89	2.80	2.06	1.69	2.51	1.89	1.55	2.31
Extremely preterm birth	2.30	1.97	2.82	1.98	1.63	2.42	1.83	1.50	2.23
Preterm birth by onset									
Spontaneous preterm birth	1.73	1.54	1.95	1.65	1.47	1.86	1.53	1.35	1.72
Indicated preterm birth	2.63	2.28	3.04	2.04	1.75	2.38	1.93	1.66	2.25
Preterm birth by number									
1 preterm birth	1.92	1.74	2.12	1.73	1.57	1.92	1.62	1.46	1.79
≥2 preterm births	3.06	2.35	3.98	2.29	1.75	2.99	2.04	1.56	2.67
Smoking^a^									
Never smoking during pregnancy	Ref						Ref		
Smoking before last pregnancy	2.10	1.81	2.43				1.64	1.41	1.90
Smoking during last pregnancy	2.29	2.14	2.44				2.07	1.93	2.23

Multivariate 1: Preterm births adjusted for maternal age at delivery, country of birth, socioeconomic status, number of births, SGA, pregestational and gestational diabetes

Multivariate 2: Variables in Multivariate 1 + maternal smoking during pregnancy

^a^: Multivariate 2 adjusted for any preterm birth

**Table 3:** Association of preterm birth and smoking with maternal coronary heart disease (n = 1,968)

	Univariate			Multivariate 1			Multivariate 2		
	HR	95 % CI		HR	95 % CI		HR	95 % CI	
CHD									
Term birth	Ref			Ref			Ref		
Any preterm birth	2.01	1.75	2.30	1.73	1.50	1.99	1.61	1.39	1.85
Preterm birth by severity									
Late preterm birth	1.79	1.50	2.15	1.59	1.33	1.90	1.49	1.24	1.78
Moderately preterm birth	2.37	1.79	3.14	2.07	1.56	2.75	1.89	1.43	2.51
Extremely preterm birth	2.30	1.77	2.98	1.86	1.39	2.48	1.72	1.29	2.29
Preterm birth by onset									
Spontaneous preterm birth	1.78	1.50	2.10	1.67	1.41	1.97	1.53	1.29	1.81
Indicated preterm birth	2.53	2.05	3.13	1.86	1.48	2.32	1.77	1.41	2.21
Preterm birth by number									
1 preterm birth	1.88	1.63	2.17	1.65	1.43	1.92	1.54	1.33	1.79
≥2 preterm births	3.64	2.55	5.19	2.58	1.79	3.71	2.31	1.61	3.33
Smoking^a^									
Never smoking during pregnancy	Ref						Ref		
Smoking before last pregnancy	1.97	1.58	2.47				1.49	1.19	1.87
Smoking during last pregnancy	2.37	2.16	2.60				2.13	1.92	2.35

Multivariate 1: Preterm birth adjusted for maternal age at delivery, country of birth, socioeconomic status, number of births, SGA, pregestational and gestational diabetes

Multivariate 2: Variables in Multivariate 1 + maternal smoking during pregnancy

^a^: Multivariate 2 adjusted for any preterm birth

**Table 4:** Association of preterm birth and smoking with maternal myocardial infarction (*n* = 720)

	Univariate			Multivariate 1			Multivariate 2		
	HR	95 % CI		HR	95 % CI		HR	95 % CI	
Term birth	Ref			Ref			Ref		
Any preterm birth	2.26	1.82	2.80	1.84	1.47	2.31	1.65	1.32	2.07
Preterm birth by severity									
Late preterm birth	2.09	1.59	2.77	1.80	1.36	2.38	1.63	1.23	2.15
Moderately preterm birth	2.61	1.49	3.80	1.98	1.24	3.18	1.73	1.08	2.78
Extremely preterm birth	2.38	1.73	3.92	1.85	1.17	2.94	1.66	1.05	2.62
Preterm birth by onset									
Spontaneous preterm birth	2.02	1.55	2.63	1.83	1.42	2.42	1.62	1.24	2.12
Indicated preterm birth	2.80	2.00	3.92	1.85	1.28	2.63	1.71	1.19	2.45
Preterm birth by number									
1 preterm birth	2.10	1.67	2.64	1.76	1.39	2.24	1.59	1.25	2.01
≥2 preterm births	4.32	2.49	7.48	2.75	1.56	4.84	2.36	1.34	4.16
Smoking^a^									
Never smoking during pregnancy	Ref						Ref		
Smoking before last pregnancy	2.45	1.71	3.52				1.78	1.23	2.58
Smoking during last pregnancy	3.48	3.00	4.05				3.04	2.58	3.57

Multivariate 1: Preterm birth adjusted for maternal age at delivery, country of birth, socioeconomic status, number of births, SGA, pregestational and gestational diabetes

Multivariate 2: Variables in Multivariate 1 + maternal smoking during pregnancy

^a^: Multivariate 2 adjusted for any preterm birth

**Table 5:** Association of preterm birth and smoking with maternal cerebrovascular event (*n* = 1,848)

	Univariate			Multivariate 1			Multivariate 2		
	HR	95 % CI		HR	95 % CI		HR	95 % CI	
Term birth	Ref			Ref			Ref		
Any preterm birth	1.94	1.69	2.23	1.81	1.56	2.09	1.68	1.46	1.95
Preterm birth by severity									
Late preterm birth	1.68	1.39	2.03	1.58	1.31	1.91	1.49	1.23	1.80
Moderately preterm birth	2.20	1.64	2.97	2.07	1.54	2.78	1.90	1.41	2.56
Extremely preterm birth	2.45	1.90	3.16	2.32	1.75	3.07	2.13	1.61	2.82
Preterm birth by onset									
Spontaneous preterm birth	1.63	1.36	1.95	1.60	1.34	1.92	1.49	1.24	1.78
Indicated preterm birth	2.63	2.13	3.24	2.25	1.80	2.81	2.12	1.70	2.65
Preterm birth by number									
1 preterm birth	1.90	1.64	2.20	1.79	1.54	2.08	1.68	1.44	1.95
≥2 preterm births	2.43	1.58	3.73	1.99	1.28	3.07	1.76	1.14	2.73
Smoking^a^									
Never smoking during pregnancy	Ref						Ref		
Smoking before last pregnancy	2.08	1.68	2.57				1.72	1.38	2.14
Smoking during last pregnancy	2.18	1.98	2.41				2.04	1.84	2.27

Multivariate 1: Preterm birth adjusted for maternal age at delivery, country of birth, socioeconomic status, number of births, SGA, pregestational and gestational diabetes

Multivariate 2: Variables in Multivariate 1 + maternal smoking during pregnancy

^a^: Multivariate 2 adjusted for any preterm birth

**Table 6:** Association of preterm birth and smoking with maternal congestive heart failure (*n* = 1,446)

	Univariate			Multivariate 1			Multivariate 2		
	HR	95 % CI		HR	95 % CI		HR	95 % CI	
Term birth	Ref			Ref			Ref		
Any preterm birth	2.99	2.34	3.83	2.50	1.92	3.24	2.27	1.74	2.94
Preterm birth by severity									
Late preterm birth	2.44	1.75	3.41	2.20	1.57	3.07	1.89	1.49	2.80
Moderately preterm birth	3.06	1.79	5.21	2.67	1.56	4.56	2.47	1.36	4.55
Extremely preterm birth	4.47	2.98	6.71	3.32	2.07	5.31	3.08	1.94	5.08
Preterm birth by onset									
Spontaneous preterm birth	2.42	1.76	3.31	2.26	1.64	3.10	2.03	1.48	2.80
Indicated preterm birth	4.29	3.02	6.10	2.99	2.03	4.39	2.74	1.87	4.03
Preterm birth by number									
1 preterm birth	2.81	2.17	3.65	2.40	1.83	3.15	2.20	1.67	2.88
≥2 preterm births	5.24	2.79	9.83	3.59	1.88	6.87	3.02	1.58	5.78
Smoking^a^									
Never smoking during pregnancy	Ref						Ref		
Smoking before last pregnancy	3.59	2.48	5.20				2.50	1.70	3.68
Smoking during last pregnancy	2.97	2.44	3.61				2.38	1.93	2.95

Multivariate 1: Preterm birth adjusted for maternal age at delivery, country of birth, socioeconomic status, number of births, SGA, pregestational and gestational diabetes

Multivariate 2: Variables in Multivariate 1 + maternal smoking during pregnancy

^a^: Multivariate 2 adjusted for any preterm birth

**Fig. 2:**
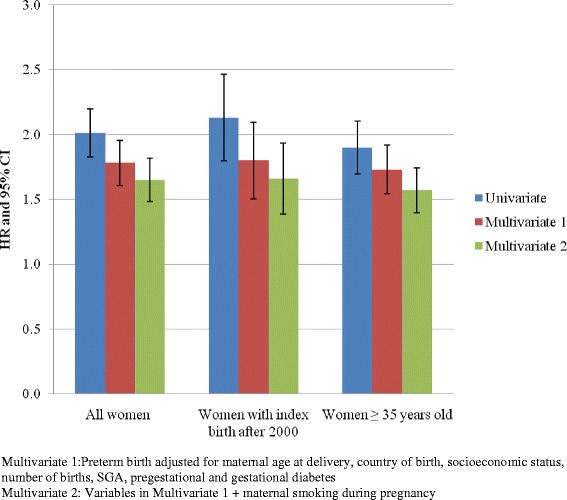
Sensitivity analysis of association of any preterm birth and maternal cardiovascular disease

Compared to non-smoking mothers, both crude and adjusted HRs of CVD (and CVD subgroups) among mothers who smoked during last pregnancy were consistently higher than that among mothers who smoked during a prior pregnancy, but not during last pregnancy (Table 2, 3, 4, 5, 6 and 7). Subgroup analyses of women who had index birth from 2001 or women ≥ 35 years of age at the commencement of follow up showed similar HRs, although 95 % CIs were wider, consistent with the smaller sample sizes (Fig. 2, Table 7).

**Table 7:** Association of any preterm birth with maternal cardiovascular disease in women with index birth from 2001 and women ≥ 35 years of age at the commencement of follow up

	Model 1			Model 2			Model 3		
	HR	95 % CI		HR	95 % CI		HR	95 % CI	
Index birth from 2001									
Any preterm birth	2.13	1.82	2.49	1.80	1.53	2.12	1.66	1.41	1.96
Smoking before last pregnancy	1.97	1.57	2.47				1.46	1.16	1.85
Smoking during last pregnancy	2.62	2.31	2.97				2.19	1.91	2.51
Women ≥ 35 years of age only									
Any preterm birth	1.94	1.74	2.10	1.70	1.52	1.90	1.59	1.43	1.77
Smoking before last pregnancy	1.91	1.61	2.68				1.53	1.29	1.83
Smoking during last pregnancy	2.25	2.09	3.03				2.07	1.91	2.23

Multivariate 1: Preterm birth adjusted for maternal age at delivery, country of birth, socioeconomic status, number of births, SGA, pregestational and gestational diabetes

Multivariate 2: Variables in Multivariate 1 + maternal smoking during pregnancy

## Discussion

This study, using linked population-based data, is the first to provide a comprehensive assessment of the relationships between preterm birth and maternal CVD later in life, taking into account the heterogeneity of preterm births. The results indicate that delivery of a preterm infant was associated with a significant increase in the risk of maternal CVD hospitaliation or death in the years following pregnancy. The associations were dose-dependent according to both the severity and number of preterm births and stronger for indicated than for spontaneous preterm birth. Consistent with our hypothesis, the associations were attenuated, but not eliminated, after accounting for maternal smoking during pregnancy. Maternal smoking during pregnancy was also associated with an increased risk of maternal CVD, regardless of whether a woman ever had a preterm birth. In particular, a higher risk estimate was observed in women who smoked during their last pregnancy compared to those who smoked during a pregnancy before the last pregnancy highlighting the benefit of smoking cessation in reducing the risk of subsequent maternal CVD. The observed associations were also independent of the established association between the hypertensive disorders of pregnancy and subsequent maternal CVD [1, 8].

Our observation of a dose-dependent association of preterm birth on maternal CVD risks is well-aligned with previous epidemiological studies. One population-based record linkage study (n = 923,686), for example, reported that having a moderate, very, and extreme preterm infant was associated with a 39 %, 157 %, and 128 % increase in the risk of subsequent maternal CVD hospitaliation or death, after accounting for smoking and other CVD risk factors [7]. In another population-based record linkage study (n = 47,908), the incidence of maternal CVD hospitalisations and morbidity in women with term births, one preterm births, and ≥ 2 preterm births were 3.5 %, 5 %, and 5.5 %, respectively [6].

In particular, our study demonstrated a stronger association with maternal CHD for indicated preterm birth. A similar gradient effect has been reported by only one previous study (n = 750,375; AHR = 1.94 [1.66–2.28] for elective preterm births compared to AHR = 1.50 [1.31 – 1.72] for spontaneous preterm birth [5]. However, it is unclear how the latter study managed elective delivery following PPROM and there was no adjustment for smoking. Differences in the aetiology of spontaneous and indicated preterm births may account for these gradient effects [5]. Indicated preterm delivery is most commonly performed as a result preeclampsia or intrauterine growth restriction [22]. In this study, we excluded the hypertensive disorders of pregnancy and accounted for SGA in the analysis. Therefore, the stronger association for indicated preterm births may reflect effects of other underlying conditions leading to planned births. On the other hand, infection is often implicated in the aetiology of spontaneous preterm labour and PPROM [23]. Inflammatory processes have been implicated in the pathogenesis of both spontaneous preterm birth and CVD [1].

A precise mechanism involved in the relationship of preterm birth and maternal CVD has not been well-established. It has been postulated that preterm birth is linked to maternal CVD later in life through shared common biological antecedents or inflammatory processes leading to the formation of atherosclerosis. For example, identifiable risk markers of preterm birth, including infection and inflammation characterised by proinflammatory cytokines, the prostaglandin cascade, and matrix metalloproteinases [24], are also related to increased risk of plaque rupture and endothelial dysfunction, leading to subsequent CVD [25]. Further, other common pathways are related to vascular lesions in the placenta, which stimulates both smooth muscle contractions and degradation of fetal membranes, two key elements in spontaneous preterm labour [24]. These lesions activate thrombin – a key component in the atherosclerotic process [26]. The underlying mechanisms involved in the relationship of cigarette smoking with elevated CVD risk, on the other hand, have been well-documented. Cigarette smoking causes a range of biological abnormalities, leading to dysfunction of the vascular system, including decreased insulin sensitivity, impaired glucose control, altered lipid profile, endothelial dysfunction, defects in coagulation and fibrinolysis, platelet dysfunction, and the generation of advanced glycation end-products [27–29].

The strengths of the current study are noteworthy. Apart from the large study population involving women of diverse cultural backgrounds, and the use of well-validated, longitudinally-linked data, the analysis was able to account for the effect of maternal smoking and other conventional CVD risk factors preceding and during pregnancy. In particular, the potential confounding effect of pregestational and pregnancy hypertension (including preeclampsia) – strong risk factors for both CVD and preterm birth was excluded. As expected, the increased risk of maternal CVD associated with either preterm birth or maternal smoking was attenuated in the multivariate models. Most previous studies used only preterm birth occurring at the index birth (first birth) to define exposure [5, 7, 30]. The current study identified all women with a history of preterm birth for analysis, thus providing a more complete ascertainment of exposure and more reliable estimate of the associations. Furthermore, the large study population provided sufficient statistical power to perform separate analyses on the specific CVD subgroups, and to differentiate the relationship according to the severity, onset, and number of preterm births.

A potential weakness of the study relates to the use of multiple datasets that do not cover the same period. There was under-ascertainment of CVD death due to the lack of cause of death data beyond 2007. However, given that only 36 deaths from CVD without prior hospital admission for CVD were identified in the first 8 years (2000 – 2007), we estimated the number of missing deaths from CVD is minimal. Another potential weakness was the delayed commencement of follow up in women who gave birth before 2000. In these women, acute cardiovascular events may have been missed, leading to an underestimate of the incidence of these events and the strength of their association with preterm birth and smoking. However, these shortcomings are not without precedent in population-based studies relying on record linkage data. In one study, for example, the follow-up only started 4–14 years after the index (first) pregnancy [31]. Furthermore, the subgroup analysis restricted to women with complete follow up provided essentially the same results. Another potential limitation is residual confounding by paternal smoking during pregnancy and other CVD risk factors before (e.g., miscarriage, maternal obesity, food and nutritional intake) and after (e.g., maternal hypertension, hyperlipidaemia) delivery, because information on these risk factors was not available in the datasets used for the present study.

## Conclusions

Based on a large retrospective cohort of women, this study provides robust evidence indicating that the association of preterm birth and future risk of maternal CVD is independent of maternal smoking and hypertensive disorder during pregnancy. This underscores the importance of smoking cessation in reducing CVD and suggests that a history of preterm delivery (especially if severe, indicated or recurrent) can identify a sizeable number of women susceptible to early onset of CVD and premature death in the years following pregnancy. Careful detailing of a woman’s reproductive life including history of preterm birth would enhance assessment of her CVD risk profile, potentially leading to more effective targeting of CVD screening, education and preventative therapies. Furthermore, pregnancy provides an opportunity for lifestyle education to encourage smoking women to quit smoking as well as for indentifying women at elevated risk of subsequent CVD.

## Abbreviations

ABS: Australian Bureau of Statistics
APDC: Admitted Patient Data Collection
CVD: Cardiovascular Disease
CHD: Coronary Heart Disease
CHF: Congestive Heart Failure
MI: Myocardial Infarction
NSW: New South Wales
PPROM: Preterm Premature Rupture of the Membranes
PDC: Perinatal Data Collection
SEIFA: Socioeconomic Indexes for Areas
SGA: Small for Gestational Age
RBDM: Registrar of Births, Deaths and Marriages
